# Association of Exposure to Persistent Organic Pollutants With Mortality Risk: An Analysis of Data From the Prospective Investigation of Vasculature in Uppsala Seniors (PIVUS) Study

**DOI:** 10.1001/jamanetworkopen.2019.3070

**Published:** 2019-04-26

**Authors:** P. Monica Lind, Samira Salihovic, Jordan Stubleski, Anna Kärrman, Lars Lind

**Affiliations:** 1 Occupational and Environmental Medicine, Department of Medical Sciences, Uppsala University, Uppsala, Sweden; 2 Inflammatory Response and Infection Susceptibility Centre, School of Medical Sciences, Örebro University, Örebro, Sweden; 3 Man-Technology-Environment (MTM) Research Center, School of Science and Technology, Örebro University, Örebro, Sweden; 4 Now with Wellington Laboratories Inc, Guelph, Ontario, Canada; 5 Cardiovascular Epidemiology, Department of Medical Sciences, Uppsala University, Uppsala, Sweden

## Abstract

**Importance:**

It has been suggested that persistent organic pollutants (POPs) are harmful to human health.

**Objective:**

To investigate if POP levels in plasma are associated with future mortality.

**Design, Setting, and Participants:**

Cohort study using data from the population-based Prospective Investigation of the Vasculature in Uppsala Seniors (PIVUS) study, collected between May 2001 and June 2004 when participants reached age 70 years. Participants were followed up for 5 years after the first examination. Mortality was tracked from age 70 to 80 years. Data analysis was conducted in January and February 2018.

**Exposures:**

Eighteen POPs identified by the Stockholm Convention, including polychlorinated biphenyls (PCBs), organochlorine pesticides, and a brominated flame retardant, were measured in plasma levels by gas chromatography–mass spectrometry.

**Main Outcomes and Measures:**

All-cause mortality.

**Results:**

The study sample initially included 992 individuals (497 [50.1%] men) aged 70 years, who were examined between 2001 and 2004. At the second examination 5 years later, 814 individuals (82.1%; 412 [50.7%] women) completed follow-up. During a follow-up period of 10.0 years, 158 deaths occurred. When updated information on POP levels at ages 70 and 75 years was associated with all-cause mortality using Cox proportional hazard analyses, a significant association was found between hexa-chloro- through octa-chloro-substituted (highly chlorinated) PCBs and all-cause mortality (except PCB 194). The most significant association was observed for PCB 206 (hazard ratio [HR] for 1-SD higher natural log–transformed circulating PCB 206 levels, 1.55; 95% CI, 1.26-1.91; *P* < .001). Following adjustment for hypertension, diabetes, smoking, body mass index, and cardiovascular disease at baseline, most associations were no longer statistically significant, but PCBs 206, 189, 170, and 209 were still significantly associated with all-cause mortality (PCB 206: adjusted HR, 1.47; 95% CI, 1.19-1.81; PCB 189: adjusted HR, 1.29; 95% CI, 1.08-1.55; PCB 170: adjusted HR, 1.24; 95% CI, 1.02-1.52; PCB 209: adjusted HR, 1.29; 95% CI, 1.04-1.60). In a secondary analysis, these associations were mainly because of death from cardiovascular diseases rather than noncardiovascular diseases. Three organochlorine pesticides, including dichlorodiphenyldichloroethylene, and the brominated flame retardant diphenyl ether 47 were also evaluated but did not show any significant associations with all-cause mortality.

**Conclusions and Relevance:**

Higher levels of highly chlorinated PCBs were associated with an increased mortality risk, especially from cardiovascular diseases. These results suggest that public health actions should be undertaken to minimize exposure to highly chlorinated PCBs.

## Key Points

**Question:**

Are elevated levels of persistent organic pollutants associated with an increased mortality risk?

**Findings:**

In a population-based cohort study of 992 individuals aged 70 to 80 years, 18 persistent organic pollutants were measured in plasma at 2 occasions. Elevated levels of highly chlorinated polychlorinated biphenyls (PCBs) were associated with increased mortality risk, mainly from cardiovascular diseases, during 10 years of follow-up.

**Meaning:**

These results indicate that public health actions should be undertaken to limit exposure to PCBs.

## Teaser

This population-based cohort study investigates if levels of persistent organic pollutants in plasma are associated with cardiovascular and all-cause mortality in participants of the Prospective Investigation of the Vasculature in Uppsala Seniors (PIVUS) study.

## Introduction

Polychlorinated biphenyls (PCBs) are a group of persistent organic pollutants (POPs) added to the Stockholm Convention in 2001 because of suspected adverse effects on wildlife and human health.^1^ Although PCBs are no longer manufactured, they are still present in the environment, and therefore humans can still be exposed to them. Exposure occurs primarily by ingesting high-fat foods, such as dairy products, eggs, and animal fats.^2^ A number of adverse health associations that are linked to cardiovascular disease (CVD) have been observed in study participants with elevated levels of PCBs, such as diabetes,^3,4,5^ obesity,^6,7,8^ hyperlipidemia,^9^ atherosclerosis,^10^ impaired myocardial left ventricular systolic function,^11^ prevalent CVD,^12^ and incident stroke.^13^

Organochlorine (OC) pesticides, such as dichlorodiphenyltrichloroethane (DDT), are another group of POPs included in the Stockholm Convention that have been banned for use in most countries because of suspected adverse health effects. Adverse cardiometabolic effects have also been reported for these OC pesticides.^14,15,16,17^ Brominated flame retardants, such as brominated diphenyl ethers (BDEs), are another group of POPs that are primarily linked to thyroid disruption,^18^ although the association varies across studies and the degree of BDE bromination.

The first studies on POPs and mortality involved occupationally exposed humans. While results from these earlier studies showed that occupational exposure to PCBs might increase all-cause mortality,^19^ to our knowledge, there is no collected evidence to support the hypothesis that workers exposed to pesticides have an increased all-cause mortality.^20,21,22^

To our knowledge, only a few population-based studies have evaluated POP levels and mortality.^23,24,25,26^ In a 1989 US-based study,^23^ no associations of exposure to DDT and/or dichlorodiphenyldichloroethylene (DDE) with all-cause mortality were found. In analyses of the US-based National Health and Nutrition Examination Survey (NHANES) study, a 2015 analysis^24^ found no significant association of the sum of PCB or OC pesticide levels with all-cause mortality in the total population. When divided into CVD and cancer mortality, the sum of PCBs showed a positive association with CVD mortality but was not associated with cancer-related death. There was also an interesting interaction between POP levels and fat mass. In a 2017 study,^25^ β-hexachlorocyclohexane, an OC pesticide not evaluated in the present study, was associated with increased all-cause mortality, while high exposure to 4 other OC pesticides, including the metabolite of DDT (p,p′DDE) and *trans*-nonachlor (TNC), were associated with increased non-cancer-related, non-CVD-related mortality. In a 2012 study also using NHANES data,^26^ circulating dioxin toxic equivalents were associated with all-cause mortality.

To further investigate the association of circulating levels of Stockholm Convention–identified POPs with mortality risk, we used data from the Prospective Investigation of the Vasculature in Uppsala Seniors (PIVUS) study,^27^ in which 18 Stockholm Convention–identified POPs were measured in participants’ plasma at ages 70 and 75 years and mortality was tracked for 10 years. The repeated measurements of POP levels from the same individuals at 2 occasions gave a more accurate estimation of POP levels and increased the power of the statistical analysis compared with previously conducted studies. The primary hypothesis tested was that increased POP levels in Swedish residents 70 years and older were associated with an increased mortality risk. As a secondary aim, we also compared the association of POP levels with cardiovascular and noncardiovascular mortality risk.

## Methods

All study participants aged 70 years living in Uppsala, Sweden, were eligible for participation in the PIVUS study.^27^ Potential study participants were randomly chosen from the registry of community inhabitants. Of the 2025 invited individuals, 1016 participated in the study between May 2001 and June 2004. They answered a questionnaire about their medical history, lifestyle habits, and regular medication at each examination. After 5 years (2006-2009), all participants were invited to a reexamination, and 826 attended (81.2%; 52 died during those 5 years). All participants were tracked from age 70 to 80 years regarding mortality (censor date 2011-2014 depending on the date of inclusion in the study). Details on measurements of traditional risk factors are given in the eMethods in the Supplement. Overall, 18 Stockholm Convention–identified POPs, including PCBs, OC pesticides, and a BDE, were measured in plasma by gas chromatography–mass spectrometry at both occasions. Data analysis was conducted in January and February 2018.

Written informed consent was obtained from all participants. The study protocol was approved by the ethical committee of the University of Uppsala, Sweden, and complies with the Declaration of Helsinki of 1975, as revised in 2008 and 2013.^28^ This cohort study followed the Strengthening the Reporting of Observational Studies in Epidemiology (STROBE) reporting guideline.

### Analysis of POPs

Plasma samples were diluted with protein-precipitating solutions and applied to Oasis Hydrophilic-Lipophilic-Balanced sorbent (Waters Corporation). The plate was rinsed with 40% methanol in water and dried prior to eluting the POPs using a 1:1 dichloromethane-to-hexane solution. Lipid degradation and water removal from the sample extracts were carried out using sulfuric acid–modified silica and sodium sulfate. Sample extracts were transferred to gas chromatography vials and evaporated to 20 µL of tetradecane. Two microliters were injected onto a DB-5MS capillary column (Agilent Technologies) using splitless injection and analyzed using a gas chromatograph (Agilent Technologies) coupled to a high-resolution magnetic sector mass spectrometer (GC-HRMS) (Micromass Autospec Ultima; Waters Corporation) operating at 10 000 or higher resolving power.

Persistent organic pollutant concentrations were quantified using isotope dilution. Overall, 143 quality control samples, including method blanks, in-house reference plasma, National Institute for Standards and Technology Standard Reference Material 1957 instrument blanks, and quantification standards, were routinely analyzed to assess the method and instrument performance. The methods showed good precision with relative SDs of most POP concentrations in quality control samples from the first (n = 95) and second (n = 48) examinations ranging from 11% to 25%. Accuracy was determined with National Institute for Standards and Technology Standard Reference Material 1957 (n = 48), and the deviation from certified POP values ranged from 3% to 27%.

Only POPs with measurements more than 75% above the level of detection were used in the further analyses. The level of detection divided by 2^0.5^ was used for measurements below the level of detection. Previous publications^29,30^ have given the levels of detection for the POPs.

### Outcome Definition

There was no loss of follow-up regarding all-cause mortality. Cardiovascular mortality was defined as having any of the *International Statistical Classification of Diseases and Related Health Problems, Tenth Revision *(*ICD-10*) codes I00 to I99. These diagnoses were obtained from death certificates or medical records and were validated by an experienced clinician (L.L.). In 3 study participants, the cause of death was not yet available at the time of the study.

### Statistical Analysis

The distribution of POPs (wet-weight data) were natural logarithm (ln) transformed to obtain normally distributed variables. The unit of each POP was set to its SD to obtain comparable associations across the POPs.

Associations of POP levels with all-cause mortality were assessed using Cox proportional hazard analysis using information on POPs (as well as covariates) from ages 70 and 75 years as updated covariates. This method increased the accuracy of the estimated POP levels and increased the power of the analysis.^31^ The follow-up period for each participant was split into 2 parts: (1) the time between 70 and 75 years and (2) the time between 75 and 80 years. For the first period, data collected for POPs and other covariates at the examination at age 70 years were used, and for the second period, data collected for POPs and other covariates at the examination at age 75 years were used. Participants who died during the first 5 years or did not attend the examination at age 75 years did not have their follow-up period split. Thus, this technique allowed a valid evaluation of study participants who participated in both examinations and those who only participated in the first examination.

Each POP was evaluated separately in 2 models. The first model was adjusted for sex and lipid levels (low-density and high-density lipoprotein cholesterol as well as serum triglyceride levels). The second model was further adjusted for hypertension, diabetes, body mass index, education, and smoking as well as prevalent CVD at age 70 years. In a secondary analysis, the same procedures as described above were repeated, but the outcome was CVD mortality or non-CVD mortality.

As further exploratory analyses, we also included interaction terms between POP levels and sex, body mass index, and smoking in separate models. We also investigated the possibility of an inverted U-shaped association, and other nonlinear associations, by using restricted-cubic spline functions with 3 knots (10th, 50th, and 90th percentiles) to model the POP data. Associations were also evaluated using a strict Bonferroni adjustment and a false discovery rate.

Stata version 14 (StataCorp) was used for the calculations. Significance tests were 2-tailed, and *P* < .05 was regarded as statistically significant. The hazard ratios (HRs) are for 1-SD change when POPs were on an ln-scale. More details on the sample as well as analytical methods are described in eMethods in the Supplement.

## Results

Basic characteristics at age 70 and 75 years are given in Table 1. Plasma levels were collected from 992 individuals (497 [50.1%] men) at age 70 years^29^ and 814 individuals (82.1%; 412 [50.7%] women) at age 75 years.^30^ Although the levels of POPs declined from age 70 to 75 years, the within-individual measurements of the different POPs at ages 70 and 75 years were highly correlated (Spearman ρ range, 0.49-0.79; *P* < .001).

**Table 1.** Basic Characteristics and Plasma Levels of POPs at Age 70 and 75 Years

Variable	Age 70 y (n = 992)	Age 75 y (n = 814)
Women, No. (%)	495 (49.9)	412 (50.7)
Education level, y in school, No. (%)		
<10	556 (56.7)	NA
10-12	175 (17.8)	NA
>12	250 (24.5)	NA
Prevalent CVD at baseline, No. (%)	142 (14.2)	NA
HDL cholesterol level, median (IQR), mg/dL	54 (46-70)	54 (46-66)
LDL cholesterol level, median (IQR), mg/dL	127 (108-151)	127 (104-155)
Serum triglycerides level, median (IQR), mg/dL	97 (80-133)	106 (80-151)
BMI, median (IQR)	26.6 (24.0-29.6)	26.4 (23.9-29.4)
Hypertension, No. (%)	715 (72.0)	662 (81.7)
Smoking, No. (%)	105 (10.5)	51 (6.2)
Diabetes, No. (%)	115 (11.6)	112 (13.8)
POPs, median (IQR), pg/mL		
PCB 74	91.4 (63.9-128.0)	56.7 (40.7-77.3)
PCB 99	90.8 (62.4-132.0)	51.3 (36.7-72.7)
PCB 118	201 (136.0-281.0)	118 (88.7-160.0)
PCB 105	32 (21.0-46.8)	30 (22.0-41.3)
PCB 153	1430 (1110.0-1850.0)	971 (743.0-1260.0)
PCB 138	819 (619.0-1120.0)	557 (404.0-753.0)
PCB 156	154 (119.0-198.0)	101 (78.7-128.0)
PCB 157	28 (21.4-37.0)	16 (1.9-22.7)
PCB 180	1170 (918.0-1488.0)	783 (625.0-965.0)
PCB 170	498 (386.0-633.0)	315 (250.0-392.0)
PCB 189	19.2 (14.6-25.8)	12.3 (1.8-16.7)
PCB 194	119 (87.6-159.0)	94 (72.0-119.0)
PCB 206	26.8 (20.8-35.2)	15.3 (2.5-20.7)
PCB 209	26.2 (19.6-34.7)	18.0 (13.3-24.0)
HCB	254 (189.0-337.0)	197 (122.0-255.0)
p,p′DDE	1860 (1020.0-3470.0)	1500 (805.0-2840.0)
TNC	140 (91.6-211.0)	97 (66.0-135.0)
BDE 47	12.6 (9.0-19.5)	4.3 (4.3-8.7)

Abbreviations: BDE, bromodiphenyl ether; BMI, body mass index (calculated as weight in kilograms divided by height in meters squared); CVD, cardiovascular disease; HCB, hexachlorobenzene; HDL, high-density lipoprotein; IQR, interquartile range; LDL, low-density lipoprotein; NA, not applicable; PCB, polychlorinated biphenyl; POPs, persistent organic pollutants; p,p′DDE, dichlorodiphenyldichloroethylene; TNC, *trans*-nonachlordane.

SI conversation factors: To convert HDL and LDL cholesterol levels to millimoles per liter, multiply by 0.0259; to convert triglyceride level to millimoles per liter, multiply by 0.0113

### All-Cause Mortality

During a follow-up period of 10.0 years (9588 person-years at risk), 158 deaths occurred. Overall, 45 deaths (28.5%) were due to CVD, 110 (69.6%) were due to non-CVD causes, and 3 (1.9%) were unclassified at the time of this study.

**Figure 1.**
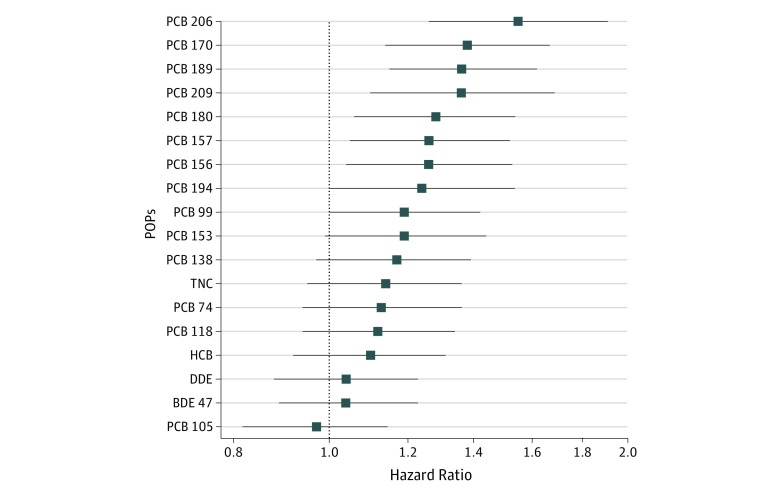
Association of Persistent Organic Pollutants (POPs) With All-Cause Mortality During 10-Year Follow-up Associations are shown for sex- and lipid-adjusted models. Updated information on POPs at ages 70 and 75 years as well as updated information on covariates were used in the calculations. Blue squares represent hazard ratios, and error bars represent 95% CIs, shown for results obtained by Cox proportional hazard models. Hazard ratios are given per 1-SD increase in natural log–transformed circulating POP values. BDE indicates bromodiphenyl ether; HCB, hexachlorobenzene; PCB, polychlorinated biphenyl; DDE, dichlorodiphenyldichloroethylene; and TNC, *trans*-nonachlordane.

After adjusting for sex and lipid levels, highly chlorinated PCB congeners (hexa-chloro-substituted, hepta-chloro-substituted, and octa-chloro-substituted) were associated with all-cause mortality (Figure 1), except for PCB 194. The most significant association was seen for PCB 206 (HR, 1.55; 95% CI, 1.26-1.91; *P* < .001). According to a strict Bonferroni adjustment, which lowered the significance level to *P* < .0028, only PCBs 206, 189, and 170 were associated with all-cause mortality. Using a false discovery rate of 5%, PCBs 206, 189, 170, 209, 180, 157, and 156 were associated with all-cause mortality. Adding examination date at age 70 years as a covariate to adjust for potential drift during the 3 years of inclusion did not have any practical impact on the results presented in Table 2.

**Table 2.** Association of POPs With All-Cause Mortality During 10 Years of Follow-up

POP	No. of Chlorine Atoms	Adjustment for Sex and Lipids		Multiple Adjustment^a^	
		HR (95% CI)^b^	*P* Value	HR (95%CI)^b^	*P* Value
PCB 206	9	1.55 (1.26-1.91)	.001	1.47 (1.19-1.81)	.001
PCB 189	7	1.36 (1.15-1.62)	.001	1.29 (1.08-1.55)	.01
PCB 170	7	1.38 (1.14-1.67)	.001	1.24 (1.02-1.52)	.03
PCB 209	10	1.36 (1.10-1.69)	.01	1.29 (1.04-1.60)	.02
PCB 180	7	1.28 (1.06-1.54)	.009	1.17 (0.96-1.42)	.12
PCB 157	6	1.26 (1.05-1.52)	.01	1.18 (0.99-1.42)	.07
PCB 156	6	1.26 (1.04-1.53)	.01	1.17 (0.96-1.42)	.13
PCB 194	8	1.24 (1.00-1.54)	.05	1.14 (0.92-1.41)	.25
PCB 99	5	1.19 (1.00-1.42)	.06	1.16 (0.97-1.38)	.11
PCB 153	6	1.19 (0.99-1.44)	.06	1.10 (0.91-1.32)	.31
PCB 138	6	1.17 (0.97-1.39)	.09	1.10 (0.92-1.32)	.28
TNC	NA	1.14 (0.95-1.36)	.16	1.07 (0.90-1.28)	.43
PCB 74	4	1.13 (0.94-1.36)	.18	1.12 (0.93-1.36)	.22
PCB 118	5	1.12 (0.94-1.34)	.22	1.15 (0.96-1.38)	.14
HCB	NA	1.10 (0.92-1.31)	.32	1.07 (0.89-1.29)	.47
PCB 105	5	0.95 (0.80-1.12)	.52	0.99 (0.83-1.18)	.91
BDE 47	NA	1.04 (0.89-1.23)	.61	1.04 (0.88-1.23)	.62
p,p′DDE	NA	1.04 (0.88-1.23)	.67	1.01 (0.85-1.20)	.90

Abbreviations: BDE, bromodiphenyl ether; HCB, hexachlorobenzene; HR, hazard ratio; NA, not applicable; PCB, polychlorinated biphenyl; POPs, persistent organic pollutants; p,p′DDE, dichlorodiphenyldichloroethylene; TNC, *trans*-nonachlordane.

^a^ Further adjustment for diabetes, hypertension, smoking, body mass index, and prevalent cardiovascular disease and education at baseline.

^b^ The HRs are given per 1-SD increase in natural log−transformed circulating POP values.

We created 2 summary measures based on our previous experience of clustering of PCBs into a highly chlorinated group (PCBs 209, 206, 194, 189, 180, 170, 157, and 156) and a less-chlorinated cluster (PCBs 153, 138, 118, 105, 99, and 74).^32^ The summary measure of the highly chlorinated PCBs was significantly associated with all-cause mortality (HR for 1-pg/mL increase in ln-transformed summary measure, 1.98; 95% CI, 1.27-3.07; *P* = .002), while the summary measure of the less-chlorinated PCBs was not significantly associated with all-cause mortality (HR for 1-pg/mL increase in the ln-transformed summary measure, 1.41; 95% CI, 0.96-2.08; *P* = .07).

The proportional hazards were evaluated by Schoenfeld residuals for each of the POPs, and the assumption was met for most POPs (including all POPs with *P* < .05 for all-cause mortality). However, as expected, the assumption was violated for POPs with HRs close to 1.00. Therefore, the exact estimate for nonsignificant POPs with HRs close to 1.00 must be taken with caution.

**Figure 2.**
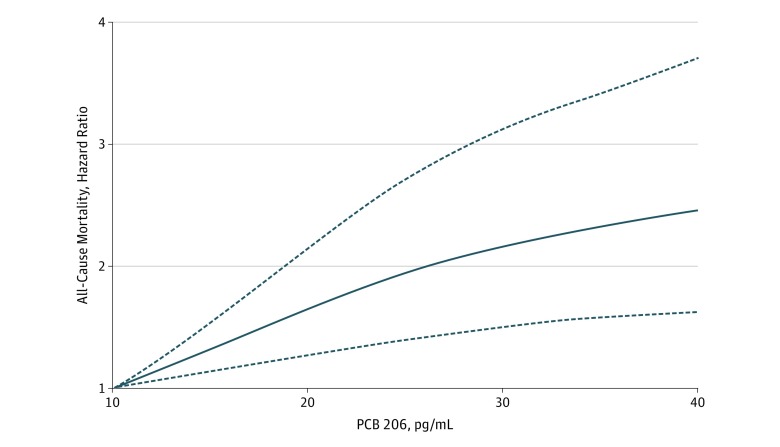
Association of Polychlorinated Biphenyl (PCB) 206 With All-Cause Mortality A restricted cubic spline model was used for PCB 206 to allow for nonlinear associations. Hazard ratios were obtained using a Cox proportional hazard analysis, adjusting for age, sex, and lipid levels. A PCB 206 level of 10 pg/mL was set as the reference. The dashed lines indicate 95% CIs. Hazard ratios are given for 1-SD increase in natural log–transformed circulating PCB 206 value.

When nonlinear associations were evaluated with a restricted cubic spline function, significant deviations from linearity were found for PCBs 206, 189, and 99 (eTable 1 in the Supplement). A slight flattening of the regression line can be seen for values greater than 30 pg/mL for PCB 206 (Figure 2). Such flattening of the concentration-HR curve was more pronounced for PCB 189, and for PCB 99, an inverse U-shaped curve could be seen (eFigure 1 and eFigure 2 in the Supplement).

When a summary measure of the dioxinlike PCBs (DLPCBs; PCBs 105, 118, 156, 157, and 189) was compared with a summary measure of the non-DLPCBs, both summary measures were associated with all-cause mortality (DLPCBs: HR for 1 pg/mL increase in ln-transformed summary measure, 1.22; 95% CI, 1.02-1.46; *P* = .03; non-DLPCBs: HR for 1 pg/mL increased in ln-transformed summary measure, 1.26; 95% CI, 1.05-1.52; *P* = .01). The 3 OC pesticides, including p,p′DDE, and BDE 47 were not significantly associated with all-cause mortality (Table 2).

Following adjustment for hypertension, diabetes, smoking, body mass index, education, and CVD at baseline, most associations were no longer statistically significant. However, PCBs 206, 189, 170, and 209 were still associated with all-cause mortality (PCB 206: adjusted HR, 1.47; 95% CI, 1.19-1.81; PCB 189: adjusted HR, 1.29; 95% CI, 1.08-1.55; PCB 170: adjusted HR, 1.24; 95% CI, 1.02-1.52; PCB 209: adjusted HR, 1.29; 95% CI, 1.04-1.60). Details from the 2 levels of adjustment are shown in Table 2.

No significant interactions between POPs and sex, body mass index, or smoking were observed regarding all-cause mortality. Baseline levels of POPs at age 70 years (omitting data from age 75 years) were not associated with all-cause mortality (eTable 2 in the Supplement). However, the highly chlorinated PCBs showed the highest HRs in this analysis.

To test for potential mixture associations, we evaluated 14 different interaction terms between POPs from different chemical groups in separate models. Because we had previously defined a less-chlorinated and a highly chlorinated cluster of PCBs with high correlations within each cluster,^32^ we only used a single representative from each of the clusters (PCB 206 and PCB 105). The evaluated interaction terms were PCB 206 × PCB 105, PCB 206 × DDE, PCB 206 × hexachlorobenzene (HCB), PCB 206 × TNC, PCB 206 × BDE 47, PCB 105 × DDE, PCB 105 × HCB, PCB 105 × TNC, PCB 105 × BDE 47, DDE × BDE 47, TNC × BDE 47, HCB × BDE 47, HCB × TNC, HCB × DDE, and TNC × DDE. None of the evaluated interaction terms were significant.

### CVD vs Non-CVD Mortality

**Figure 3.**
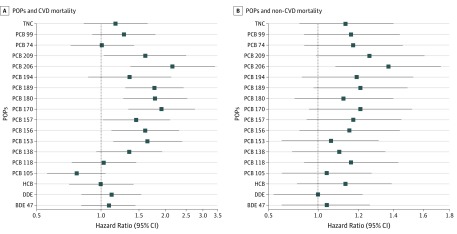
Association of Persistent Organic Pollutants (POPs) With Cardiovascular Disease (CVD) and Non-CVD Mortality, for 10-Year Follow-up Associations are shown for sex- and lipid-adjusted models. Updated information on POPs at ages 70 and 75 years as well as updated information on the covariates were used in the calculations. Hazard ratios and 95% CIs are shown for results obtained using Cox proportional hazard models. Hazard ratios are given per 1-SD increase in natural log–transformed circulating POP values. BDE indicates bromodiphenyl ether; HCB, hexachlorobenzene; PCB, polychlorinated biphenyl; DDE, dichlorodiphenyldichloroethylene; and TNC, *trans*-nonachlordane.

The HRs were larger and the CIs smaller for the highly chlorinated PCBs with CVD mortality vs non-CVD mortality as the outcome, despite the fact that the number of non-CVD mortality cases was more than double the number of CVD mortality cases (Figure 3). Only PCB 206 was associated with non-CVD mortality (HR, 1.37; 95% CI, 1.08-1.73; *P* = .01) following multiple adjustments, while all PCBs evaluated with a congener number of 156 and higher were significant when adjusted for sex and lipid levels, except for PCB 194 (eTable 3 in the Supplement). Polychlorinated biphenyl 206 was more closely associated with CVD mortality than with non-CVD mortality (CVD mortality: HR, 2.16; 95% CI, 1.37-3.40; *P* < .001; non-CVD mortality: HR, 1.37; 95% CI, 1.08-1.73; *P* = .01).

## Discussion

This study showed plasma concentrations of highly chlorinated PCBs to be associated with all-cause mortality. This was most evident in the study participants dying from CVD. Circulating levels of the 3 OC pesticides, including p,p′DDE, and BDE 47 were not associated with mortality.

Associations of POPs with mortality have previously been evaluated in occupational exposure studies in which no measurements of circulating levels were performed. In a large study of 24 865 capacitor-manufacturing workers who had been exposed to PCBs,^19^ the short-term workers showed an increased total mortality rate of 17%, but the long-term workers did not. Among short-term workers, the risk of cardiovascular mortality and cancer mortality were increased compared with long-term workers (14% and 20%, respectively).

Globally, DDT is a widely used pesticide. In a study of 4552 male workers exposed to DDT during antimalarial operations in Sardinia, Italy,^33^ no increased overall mortality was found during decades of follow-up. There was a 5% increase in cancer mortality but a 29% reduction in CVD mortality. No increase in mortality was seen among 1999 individuals working outdoors as part of an insecticide application program from 1935 to 1996 in Australia compared with controls.^21^ In a study of 62 960 registered pesticide users in the United Kingdom, the mortality rate was lower compared with the expected rate in the country.^22^ Also, in the Agricultural Health Study,^20^ which examined a cohort of 89 656 pesticide applicators and their spouses in North Carolina and Iowa, a lower mortality rate was found.

Thus, in the occupational exposure studies, no detrimental associations of pesticides with mortality were found, while some evidence of an association of PCB exposure with mortality has been reported. In these studies, the exposures would be assumed to be considerably higher than what was seen in the general population, but because nonlinear relations have been reported for POPs,^34^ it does not rule out the possibility of different associations in the general population. In 2 studies on capacitor-manufacturing workers,^35,36^ PCB levels that are 5-fold to 60-fold higher than in the present study were reported. Occupational studies also have a problem with the healthy worker effect (ie, participants have a lower mortality risk than the general population), which also might have affected the results.

In a population-based study of 900 individuals conducted in the 1970s, when DDT use had peaked, no significant association of high-serum DDT/p,p′DDE levels with overall mortality or cancer mortality was found for a 10-year follow-up period.^23^ In a study investigating the association of POPs with mortality using NHANES data,^24^ no significant associations of the sum of PCB or OC pesticide levels with all-cause mortality were seen in the total population. When divided into CVD and cancer mortality, the sum of PCBs showed a positive association with CVD mortality but no significant association with cancer death. When each separate PCB was evaluated, as in the present study, no clear association was found between the degree of chlorination and CVD mortality. A major finding from the NHANES study suggested an association of levels of POPs with fat mass, in that individuals with a fat mass greater than 75% showed an inverse association of the sum of PCBs with all-cause mortality.^24^ The levels of PCBs and OC pesticides were similar in the the NHANES 2003-2004 examination subsample of individuals 70 years and older as well as in the PIVUS data for individuals aged 70 years (collected 2001-2004).^32^

In a separate analysis of NHANES data,^25^ no association was found between PCBs and mortality. However, in that study, PCB levels were only evaluated as the sum of PCBs or the dioxin toxic equivalents of the DLPCBs and not as individual PCBs. One of the evaluated OC pesticides, β-hexachlorocyclohexane, a compound not evaluated in the present study, was associated with increased all-cause mortality, while high exposure to 4 other OC pesticides, including p,p′DDE and TNC, was associated with increased noncancer, non-CVD mortality.

An important difference between the NHANES-based studies and the present study is that the most highly chlorinated PCBs evaluated in the present investigation (PCBs 206, 194, 209, and 189) were not evaluated in the studies based on NHANES data, and it was among those highly chlorinated PCBs that the most powerful associations with mortality were found in the present study. Thus, the NHANES-based studies might have underestimated the association of PCBs with mortality. Furthermore, based on our experience of different associations for less-chlorinated and highly chlorinated PCBs,^7,8^ we evaluated the PCBs separately and found profound differences in their associations with mortality; less-chlorinated PCBs were not significantly associated with mortality, while the highly chlorinated PCBs were. This might explain why other studies using the sum of PCBs have not found concordant results.

Another reason not to use a summary measurement of the PCBs is that PCBs do not share the same mode of action. Dioxinlike PCBs bind to the aryl hydrocarbon receptor, which may induce a number of adverse health associations. However, in the present study, there was no clear indication that the DLPCBs were more strongly associated with mortality than non-DLPCBs. This outcome could be anticipated from a previous study in which high toxin equivalents were associated with all-cause mortality.^26^ Unfortunately, the PCB with the highest binding to aryl hydrocarbon receptor (PCB 126), while measurable at age 70 years, had declined to the point that it was below the limit of detection at age 75 years in almost all study participants. Therefore, it could not be evaluated in the present study using measurements at both examinations. However, a separate analysis using only data from age 70 years did not show any significant association of PCB 126 levels with all-cause mortality, and when a summary measure of DLPCBs was compared with a summary measure of non-DLPCBs, DLPCBs were not more closely associated with all-cause mortality than non-DLPCBs. Taken with our other results, it is therefore likely that other mechanisms besides aryl hydrocarbon receptor binding are involved in the associations observed between highly chlorinated PCBs and mortality in the present study.

In the present study, the associations found for highly chlorinated PCBs were stronger for cardiovascular than for noncardiovascular mortality, despite the fact that there were fewer deaths due to CVD. We and others have previously shown that POPs might adversely affect important cardiovascular risk factors, such as hypertension, obesity, lipid levels, and diabetes,^3,4,5,6,7,8,9,37^ and might also affect atherosclerosis.^10^ Our novel data on cardiovascular mortality fits well with previous publications on a cross-sectional association of POPs with CVD in NHANES data.^12^ It also fits with other prospective studies in which PCB exposure has been evaluated by other means than by circulating levels.^38,39,40,41,42^ Because the PIVUS study is primarily a cardiovascular cohort, we do not have data on cancer. Approximately 80% of non-CVD deaths in Sweden are due to cancer, so most non-CVD mortality is cancer related. Unfortunately, this could not be studied in detail in the present study.

### Strengths and Limitations

The major advantage of the present study is the use of data on POPs measured at 2 occasions. Repeated measurements will give a more accurate picture of the exposure and also increase the power when used in a statistical analysis. This was evident when we performed an analysis using only the baseline data from individuals aged 70 years, omitting the data collected at age 75 years. In that case, no significant associations with all-cause mortality were seen, but highly chlorinated PCBs had the highest HRs in that analysis as well. We have previously reported the trends of these POPs during the 5-year period in detail^30^ and shown that most POP levels declined by 30% to 40%, but we could not find any association among the PCBs of the degree of decline in levels with mortality.

Although the levels of PCBs are declining because of the ban on the use of these chemicals, we are still exposed to PCBs owing to their persistence in nature and accumulation in the food chain. Thus, today’s PCB exposure could be assessed by dietary history,^43^ as elevated levels of PCBs are still found in meat and fish. Thus, from a public health perspective, actions should be taken to prevent further exposure to PCBs from these sources.

Levels of PCBs are generally associated with each other as well as with the OC pesticides evaluated in the present study. We therefore chose to regard *P* < .05 as significant, since a Bonferroni adjustment for 18 contaminants that are closely related to each other is an overadjustment. An alternative is using the more liberal false discovery rate approach to adjust for multiple tests. Using a false discovery rate of 5%, all highly chlorinated PCBs evaluated (PCB 156-209) were associated with all-cause mortality. However, it should be pointed out that the *P* value for PCB 206 regarding all-cause mortality (the primary analysis) would also be significant following a strict Bonferroni adjustment.

A cluster analysis performed in the PIVUS study^32^ has shown that less-chlorinated PCBs form a single cluster and highly chlorinated PCBs form another cluster, an observation that fits well with the present observation that it is mainly highly chlorinated PCBs that are associated with mortality. The high associations between PCBs within that cluster make it difficult to evaluate if it is a specific compound that is associated with mortality or rather common associations for all PCBs in the highly chlorinated cluster. The finding that a summary measure of highly chlorinated PCBs was associated with an increased mortality risk, while less-chlorinated PCBs were not, further stresses the importance of regarding highly chlorinated PCBs as a group and not only as single compounds.

We could not confirm previous observations that obesity^24^ or smoking^44^ modify the association of POPs with mortality. However, we did find evidence for nonlinear associations of PCBs 206, 189, and 99 with all-cause mortality. This association was most pronounced for PCB 99, for which an inverted U-shaped association was seen. Such nonmonotonic associations of POPs have been presented previously.^34^

It is common to normalize POP levels for lipid concentration. However, since both animal and human experiments^9,45^ have shown that high POP levels are associated with changes in lipids over time, we preferred to adjust for lipid levels in the statistical analysis, as previously advocated by Gaskins and Schisterman^46^ based on statistical considerations.

We have studied a very homogeneous sample of Swedish residents older than 70 years, and therefore studies in other populations are needed to confirm our findings. Furthermore, since only 0.5 mL of plasma was available for analysis, we could only detect POPs with the highest concentrations, so we lack data on important POPs, such as dioxins and furans. Another limitation is that the number of CVD mortalities is small and that misclassifications of the cause of death could appear, although all death certificates have been validated by an experienced clinician (L.L.). However, any misclassification would only reduce our chance of finding a difference between CVD and non-CVD mortality.

## Conclusions

In conclusion, higher levels of highly chlorinated PCBs were associated with an increased mortality risk, especially from CVDs. Our results suggest that public health actions should be taken to limit exposure to highly chlorinated PCBs.

## Notes

Supplement. **eMethods. **Investigation and Summary Measurements **eTable 1. **Hazard Ratios and 95% CIs for the Nonlinear Spline Term in the Analysis of Potential Nonlinear Associations of POPs With All-Cause Mortality **eTable 2. **Association of Persistent Organic Pollutants (POPs) at Age 70 Years With All-Cause Mortality **eTable 3. **Association of Persistent Organic Pollutants (POPs) With Cardiovascular (CV) and Non-CV Mortality During 10 Years of Follow-up **eFigure 1. **Association of Levels of PCB 99 With All-Cause Mortality **eFigure 2. **Association of Levels of PCB 189 With All-Cause Mortality **eReferences**
