# Supplementary material for: Association of Exposure to Persistent Organic Pollutants With Mortality Risk: An Analysis of Data From the Prospective Investigation of Vasculature in Uppsala Seniors (PIVUS) Study
Source: JAMA Netw Open. 2019 Apr 26;2(4):e193070. doi: 10.1001/jamanetworkopen.2019.3070 (PMC6487572; doi:10.1001/jamanetworkopen.2019.3070)
Supplement: Supplement. — eMethods. Investigation and Summary Measurements eTable 1. Hazard Ratios and 95% CIs for the Nonlinear Spline Term in the Analysis of Potential Nonlinear Associations of POPs With All-Cause Mortality eTable 2. Association of Persistent Organic Pollutants (POPs) at Age 70 Years With All-Cause Mortality eTable 3. Association of Persistent Organic Pollutants (POPs) With Cardiovascular (CV) and Non-CV Mortality During 10 Years of Follow-up eFigure 1. Association of Levels of PCB 99 With All-Cause Mortality eFigure 2. Association of Levels of PCB 189 With All-Cause Mortality eReferences [file jamanetwopen-2-e193070-s001.pdf]

Supplementary Online Content

Lind PM, Salihovic S, Stubleski J, Kärrman A, Lind L. Association of exposure to persistent organic pollutants with mortality risk: an analysis of data from the Prospective Investigation of Vasculature in Uppsala Seniors (PIVUS) study. *JAMA Netw Open*. 2019;2(4):e193070. doi:10.1001/jamanetworkopen.2019.3070

eMethods. Investigation and Summary Measurements
eTable 1. Hazard Ratios and 95% CIs for the Nonlinear Spline Term in the Analysis of Potential Nonlinear Associations of POPs With All-Cause Mortality
eTable 2. Association of Persistent Organic Pollutants (POPs) at Age 70 Years With All-Cause Mortality
eTable 3. Association of Persistent Organic Pollutants (POPs) With Cardiovascular (CV) and Non-CV Mortality During 10 Years of Follow-up
eFigure 1. Association of Levels of PCB 99 With All-Cause Mortality
eFigure 2. Association of Levels of PCB 189 With All-Cause Mortality
eReferences

This supplementary material has been provided by the authors to give readers additional information about the work.

© 2019 Lind PM et al. *JAMA Network Open.*

eMethods. Investigation and Summary Measurements

Investigation

All individuals were investigated in the morning after an over-night fast. Blood was drawn, and the serum and plasma were separated and frozen in -80C for later analysis of POPs. Glucose and lipids were measured on fresh samples the same day by conventional techniques at the clinical chemistry laboratory at the hospital. Blood pressure was measured by a mercury sphygmomanometer in the supine position after 30 min rest. BMI was calculated as the body weight divided by squared height. Smoking was recorded as current smoker. Education level was grouped into <10 years, 10-12 and >12 years in school.

Hypertension was defined as antihypertensive treatment or blood pressure >= 140/90 mmHg. Diabetes was defined as a diagnosis of diabetes or fasting plasma glucose >= 7.0 mmol/l.

All investigations were performed in the same fashion at both ages 70 and 75.

Two different summary measurements of PCB concentrations were created based on the number of chlorine atoms, the sum of less-chlorinated PCBs (PCBs 153, 138, 118, 105, 99, 74), and highly chlorinated PCBs (PCBs 209, 206, 194, 189, 180, 170, 157 and 156). These two summary measurements were obtained by adding the wet weight (pg/ml) concentrations. The grouping into less and highly-chlorinated PCB were based on our previous findings of clustering of PCBs into two distinct groups [1], and has been used in previous publications.

We also created two different summary measurements of PCB concentrations-based dioxin-like activity, dioxin-like PCBs (PCBs 105, 118, 156, 157 and 189) and non-dioxin like PCBs (PCBs 74, 99, 153, 138, 180, 170, 194, 206, 209). Also, these two summary measurements were obtained by adding the wet weight (pg/ml) concentrations. We also calculated the total toxic equivalence (TEQ) value for the dioxin-like PCBs according to Van den Berg et al [2].

© 2019 Lind PM et al. *JAMA Network Open.*

eTable 1. Hazard Ratios and 95% CIs for the Nonlinear Spline Term in the Analysis of Potential Nonlinear Associations of POPs With All-Cause Mortality

Restricted cubic splines with three knots (10th, 50th and 90th percentile were used). A p-value<0.05 indicates a non-linear relationship.

variable	HR (95%CI)	p-value
PCB74	0.99 (0.98-1.01)	0.23
PCB99	0.98 (0.97-0.99)	0.01
PCB118	1.01 (0.99-1.03)	0.30
PCB105	1.01 (0.99-1.03)	0.31
PCB153	1.01 (0.99-1.04)	0.65
PCB138	1.01 (0.99-1.04)	0.66
PCB156	1.01 (0.99-1.04)	0.61
PCB157	0.98 (0.96-1.01)	0.10
PCB180	1.01 (0.99-1.04)	0.30
PCB170	1.01 (0.99-1.04)	0.50
PCB189	0.96 (0.94-0.99)	0.009
PCB194	1.00 (0.99-1.01)	0.51
PCB206	0.97 (0.94-0.99)	0.03
PCB209	0.97 (0.94-1.01)	0.15
HCB	1.01 (0.99-1.03)	0.40
DDE	1.01 (0.99-1.04)	0.23
TNC	1.01 (0.99-1.04)	0.60
BDE47	0.94 (0.87-1.01)	0.11

Abbreviations: HR, hazard ratio; CI, confidence interval; PCB, polychlorinated biphenyls; HCB, hexachlorobenzene; *p,p*’-DDE, 2,2-bis (4-chlorophenyl)-1,1-dichloroethene; BDE, bromodiphenyl ether; TNC, *trans*-nonachlordane

© 2019 Lind PM et al. *JAMA Network Open.*

eTable 2. Association of Persistent Organic Pollutants (POPs) at Age 70 Years With All-Cause Mortality

Associations are shown for two levels of adjustment, sex and lipids (LDL and HDL cholesterol and serum triglycerides), as well as further adjustment for diabetes, hypertension, smoking, BMI, education level and prevalent cardiovascular disease at baseline.

	Adjustment for sex and lipids		Multiple adjustment	
variable	HR (95%CI)	P-value	HR (95%CI)	P-value
BDE47	0.83 (0.68-1.01)	0.07	0.84 (0.69-1.03)	0.09
PCB105	0.92 (0.77-1.08)	0.31	0.94 (0.79-1.12)	0.47
PCB 74	0.92 (0.78-1.08)	0.31	0.90 (0.75-1.07)	0.22
PCB 118	0.92 (0.78-1.08)	0.31	0.92 (0.78-1.10)	0.38
HCB 70	0.93 (0.78- 1.10)	0.38	0.89 (0.75-1.07)	0.22
PCB 157	0.96 (0.81-1.13)	0.63	0.89 (0.75-1.05)	0.17
TNC	0.97 (0.82-1.14)	0.69	0.92 (0.78-1.08)	0.31
PCB99	0.97 (0.82-1.14)	0.69	0.95 (0.80-1.11)	0.51
PCB138	0.97 (0.82-1.14)	0.69	0.92 (0.78-1.09)	0.33
PCB153	0.98 (0.83-1.15)	0.80	0.93 (0.79-1.09)	0.37
DDE	0.99 (0.84-1.16)	0.88	0.97 (0.82-1.14)	0.69
PCB156	1.01 (0.85-1.19)	0.93	0.96 (0.81-1.13)	0.61
PCB189	1.02 (0.87-1.20)	0.79	0.96 (0.80-1.15)	0.65
PCB180	1.03 (0.87-1.22)	0.70	0.97 (0.81-1.15)	0.70
PCB170	1.05 (0.88-1.24)	0.61	0.97 (0.81-1.16)	0.71
PCB194	1.07 (0.90-1.27)	0.47	1.02 (0.86-1.22)	0.79
PCB209	1.11 (0.94-1.32)	0.23	1.07 (0.90-1.28)	0.45
PCB206	1.13 (0.95-1.35)	0.16	1.09 (0.91-1.31)	0.35

Abbreviations: HR, hazard ratio; CI, confidence interval; PCB, polychlorinated biphenyls; HCB, hexachlorobenzene; *p,p'*-DDE, 2,2-bis (4-chlorophenyl)-1,1-dichloroethene; BDE, bromodiphenyl ether; TNC, *trans*-nonachlordane

© 2019 Lind PM et al. *JAMA Network Open.*

eTable 3. Association of Persistent Organic Pollutants (POPs) With Cardiovascular (CV) and Non-CV Mortality During 10 Years of Follow-up

Associations are shown for two levels of adjustment, sex and lipids (LDL and HDL cholesterol and serum triglycerides), as well as further adjustment for diabetes, hypertension, smoking, BMI, and prevalent cardiovascular disease at baseline. Updated information on POPs at ages 70 and 75 is used in the calculations as well as updated information on the covariates.

**Cardiovascular mortality**				
	Adjustment for sex and lipids		Multiple adjustment	
	HR<br>(95%CI)	P-value	HR<br>(95%CI)	P-value
PCB189	1.78 (1.3-2.43)	0.001	1.55 (1.11-2.18)	0.01
PCB170	1.92 (1.34-2.75)	0.001	1.58 (1.08-2.33)	0.02
PCB180	1.79 (1.27-2.53)	0.001	1.44 (1-2.09)	0.05
PCB206	2.16 (1.37-3.4)	0.001	1.76 (1.15-2.71)	0.010
PCB153	1.65 (1.14-2.4)	0.010	1.41 (0.97-2.03)	0.07
PCB156	1.61 (1.12-2.32)	0.01	1.37 (0.93-2.01)	0.11
PCB209	1.61 (1.03-2.5)	0.04	1.26 (0.83-1.89)	0.28
PCB157	1.46 (1.02-2.1)	0.04	1.31 (0.92-1.87)	0.14
PCB138	1.36 (0.95-1.93)	0.09	1.23 (0.88-1.72)	0.22
PCB105	0.77 (0.56-1.05)	0.10	0.91 (0.66-1.26)	0.58
PCB99	1.28 (0.91-1.8)	0.15	1.25 (0.9-1.75)	0.18
PCB194	1.36 (0.87-2.13)	0.17	1.02 (0.69-1.52)	0.90
TNC	1.17 (0.83-1.65)	0.37	1.17 (0.83-1.64)	0.36
DDE	1.12 (0.81-1.54)	0.49	1.12 (0.81-1.57)	0.50
BDE47	1.09 (0.81-1.45)	0.58	1.06 (0.78-1.43)	0.72
PCB118	1.03 (0.73-1.46)	0.85	1.19 (0.85-1.67)	0.32
PCB74	1.01 (0.72-1.43)	0.95	1.07 (0.76-1.51)	0.70
HCB	1 (0.71-1.42)	0.99	1.08 (0.77-1.53)	0.65
**Non-cardiovascular mortality**				
	Adjustment for sex and lipids		Multiple adjustment	
	HR<br>(95%CI)	P-value	HR<br>(95%CI)	P-value
PCB206	1.37 (1.08-1.73)	0.010	1.34 (1.05-1.70)	0.02
PCB209	1.26 (0.99-1.61)	0.06	1.27 (0.99-1.62)	0.07
PCB189	1.21(0.98-1.49)	0.07	1.22 (0.98-1.50)	0.07
PCB170	1.21 (0.96-1.52)	0.10	1.16 (0.92-1.48)	0.21
PCB157	1.17 (0.95-1.45)	0.15	1.14 (0.92-1.41)	0.24
PCB74	1.17 (0.94-1.46)	0.16	1.16 (0.93-1.44)	0.19
PCB194	1.19 (0.93-1.53)	0.17	1.19 (0.92-1.54)	0.20
PCB99	1.16 (0.94-1.44)	0.17	1.13 (0.92-1.40)	0.25
PCB118	1.16 (0.94-1.43)	0.18	1.17 (0.94-1.46)	0.15
PCB156	1.15 (0.92-1.44)	0.23	1.11 (0.88-1.39)	0.38
TNC	1.13 (0.92-1.40)	0.25	1.11 (0.89-1.37)	0.35

© 2019 Lind PM et al. *JAMA Network Open.*

HCB	1.13 (0.91-1.39)	0.27	1.1 (0.89-1.36)	0.38
PCB180	1.12 (0.9-1.40)	0.32	1.08 (0.86-1.36)	0.51
PCB138	1.1 (0.89-1.35)	0.39	1.06 (0.86-1.31)	0.58
PCB153	1.06 (0.85-1.31)	0.60	1.01 (0.82-1.26)	0.89
PCB105	1.04 (0.85-1.27)	0.72	1.07 (0.87-1.31)	0.54
BDE47	1.04 (0.85-1.26)	0.73	1.03 (0.85-1.25)	0.79
DDE	1 (0.82-1.22)	0.99	0.98 (0.8-1.20)	0.83

Abbreviations: HR, hazard ratio; CI, confidence interval; PCB, polychlorinated biphenyls; HCB, hexachlorobenzene; *p,p'*-DDE, 2,2-bis (4-chlorophenyl)-1,1-dichloroethene; BDE, bromodiphenyl ether; TNC, *trans*-nonachlordane

© 2019 Lind PM et al. *JAMA Network Open.*

eFigure 1. Association of Levels of PCB 99 With All-Cause Mortality

Figure legend: Hazard ratios regarding all-cause mortality are given using Cox proportional hazard analysis adjusting for age, sex and lipids. A cubic restricted spline model was used for PCB99to allow for non-linear associations. A PCB99 level of 50 is set as the referent (HR=1.0). The dashed lines indicate 95% confidence limits.

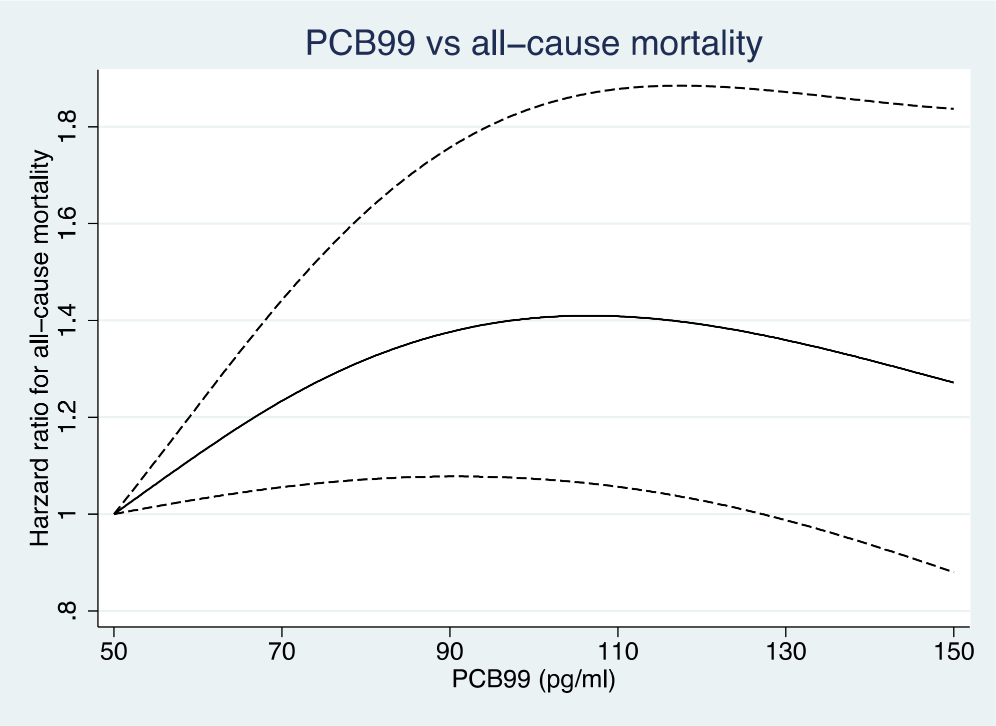

Abbreviations: PCB, polychlorinated biphenyls

© 2019 Lind PM et al. *JAMA Network Open.*

eFigure 2. Association of Levels of PCB 189 With All-Cause Mortality

Figure legend: Hazard ratios regarding all-cause mortality are given using Cox proportional hazard analysis adjusting for age, sex and lipids. A cubic restricted spline model was used for PCB189 to allow for non-linear associations. A PCB189 level of 5 is set as the referent (HR=1.0). The dashed lines indicate 95% confidence limits.

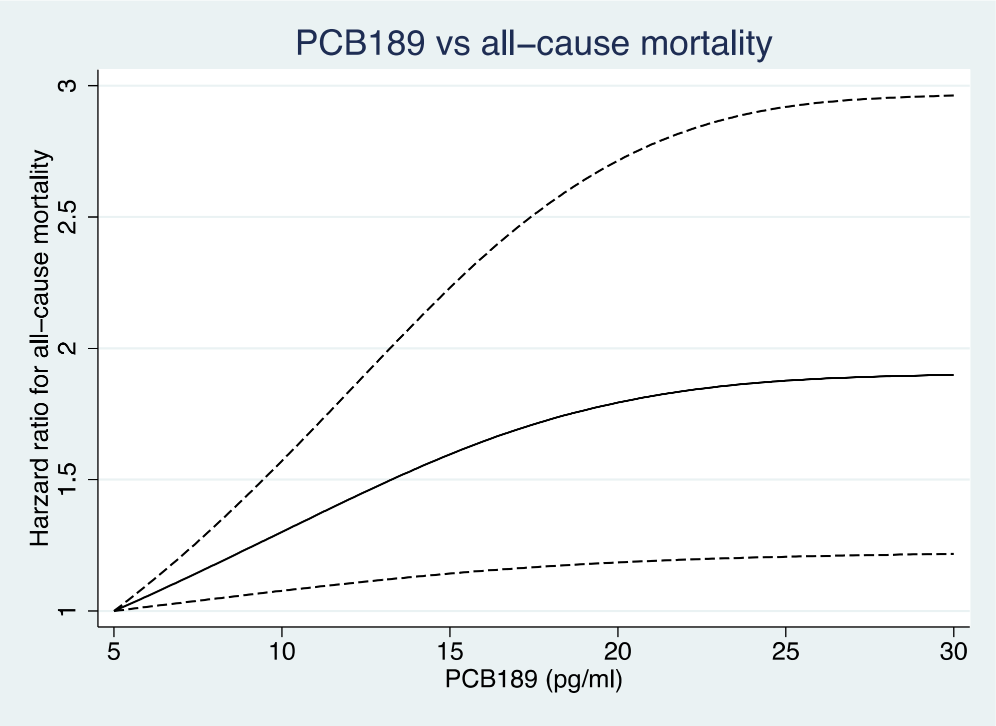

Abbreviations: PCB, polychlorinated biphenyls

© 2019 Lind PM et al. *JAMA Network Open.*

eReferences

1. Lampa E, Lind L, Hermansson AB, Salihovic S, van Bavel B, Lind PM. An investigation of the co-variation in circulating levels of a large number of environmental contaminants. J Expo Sci Environ Epidemiol 2012;**22**(5):476-82 doi: 10.1038/jes.2012.41[published Online First: Epub Date]|.
2. Van den Berg M, Birnbaum L, Bosveld AT, et al. Toxic equivalency factors (TEFs) for PCBs, PCDDs, PCDFs for humans and wildlife. Environ Health Perspect 1998;**106**(12):775-92 doi: 10.1289/ehp.98106775[published Online First: Epub Date]|.

© 2019 Lind PM et al. *JAMA Network Open.*
